# Sulforaphane has opposing effects on TNF-alpha stimulated and unstimulated synoviocytes

**DOI:** 10.1186/ar4059

**Published:** 2012-10-27

**Authors:** Athanassios Fragoulis, Jendrik Laufs, Susanna Müller, Ulf Soppa, Stephanie Siegl, Lucy Kathleen Reiss, Mersedeh Tohidnezhad, Christian Rosen, Klaus Tenbrock, Deike Varoga, Sebastian Lippross, Thomas Pufe, Christoph Jan Wruck

**Affiliations:** 1 Department of Anatomy and Cell Biology, University Hospital Aachen, RWTH Aachen University, Wendlingweg 2, 52074 Aachen, Germany; 2 Institute of Pharmacology and Toxicology, University Hospital Aachen, RWTH Aachen University, Wendlingweg 2, 52074 Aachen, Germany; 3 Department of Paediatric and Adolescent Medicine, University Hospital Aachen, RWTH Aachen University, Pauwelsstrasse 30, 52074 Aachen, Germany; 4 Department of Trauma Surgery, University Hospital of Schleswig-Holstein UK-SH, Campus Kiel, Arnold-Heller Strasse 3, 24105 Kiel, Germany

## Abstract

**Introduction:**

Rheumatoid arthritis (RA) is characterized by progressive inflammation associated with rampantly proliferating synoviocytes and joint destruction due to oxidative stress. Recently, we described nuclear factor erythroid 2-related factor 2 (Nrf2) as a major requirement for limiting cartilage destruction. NF-κB and AP-1 are the main transcription factors triggering the inflammatory progression in RA. We used sulforaphane, an isothiocyanate, which is both an Nrf2 inducer and a NF-κB and AP-1 inhibitor.

**Methods:**

Cultured synoviocytes were stimulated with sulforaphane (SFN) with or without TNF-α pre-treatment. NF-κB, AP-1, and Nrf2 activation was investigated via dual luciferase reporter gene assays. Matrix metalloproteinases (MMPs) were measured via zymography and luminex technique. Cytokine levels were detected using ELISA. Cell viability, apoptosis and caspase activity were studied. Cell proliferation was analysed by real-time cell analysis.

**Results:**

SFN treatment decreased inflammation and proliferation dose-dependently in TNF-α-stimulated synoviocytes. SFN did not reduce MMP-3 and MMP-9 activity or expression significantly. Interestingly, we demonstrated that SFN has opposing effects on naïve and TNF-α-stimulated synoviocytes. In naïve cells, SFN activated the cytoprotective transcription factor Nrf2. In marked contrast to this, SFN induced apoptosis in TNF-α-pre-stimulated synoviocytes.

**Conclusions:**

We were able to show that SFN treatment acts contrary on naïve and inflammatory synoviocytes. SFN induces the cytoprotective transcription factor Nrf2 in naïve synoviocytes, whereas it induces apoptosis in inflamed synoviocytes. These findings indicate that the use of sulforaphane might be considered as an adjunctive therapeutic strategy to combat inflammation, pannus formation, and cartilage destruction in RA.

## Introduction

Rheumatoid arthritis (RA) is an inflammatory autoimmune disease, in which the proinflammatory transcription factors nuclear factor kappa-light-chain-enhancer of activated B cells (NF-κB) and activator protein-1 (AP-1) are activated by inflammatory cytokines, which in turn upregulate the expression of these cytokines, thereby assembling a positive feedback loop perpetuating inflammation [1,2]. Moreover, TNF-α induces cell proliferation in synovial cells and triggers the generation of pannus tissue [3,4].

Searching for agents that are potentially beneficial in RA, we tested sulforaphane (SFN) in an *in vitro *model of RA. SFN is known as a potent inducer of the transcription factor nuclear factor erythroid 2-related factor 2 (Nrf2), which upregulates a battery of protective enzymes [5]. Moreover, it has been shown that SFN suppresses proliferation and induces apoptosis in various cancer cells [6]. Recently, we provided strong evidence that oxidative stress is significantly involved in cartilage degradation in experimental arthritis, indicating that Nrf2 activation is a major requirement for limiting cartilage destruction [7,8]. Hinoi *et al*. provided first evidence that Nrf2 is a negative regulator of chondrocyte differentiation during embryogenesis and postnatal development [9]. On the other hand, Nrf2 seemed to protect differentiated chondrocytes in a mouse model of RA [8].

In the present study, we used the human synoviocyte cell lines HSE and K4IM, which were stimulated with TNF-α to mimic a state of inflammation. We were able to show that SFN selectively induces apoptosis in TNF-α pre-stimulated but not in unstimulated synoviocytes. In addition, SFN stimulates Nrf2 activity and renders unstimulated synoviocytes against oxidative stress. These findings indicate that treatment of RA patients with SFN might inhibit inflammation and pannus formation while preserving healthy tissue.

## Materials and methods

### Material

RPMI 1640 medium with 2 mM glutamine was obtained from PAA Laboratories, Pasching, Austria. Sulforaphane (SFN) was obtained from Sigma-Aldrich Chemical Company, Munich, Germany. All other chemicals were of the highest quality commercially available.

### Cell culture

The human synoviocyte cell line HSE was obtained from Oligene, Berlin, Germany. These cells were produced by immortalisation of primary human synovial fibroblasts from a confirmed RA patient. Immortalisation was performed using a pGEM vector containing a SV40 Tag-encoding DNA fragment [10]. The immortalised human synoviocyte cell line K4IM was a generous gift from Christian Kaps (Charité, Berlin, Germany). These cells originate from synovial tissue of a 41-year-old male donor suffering from a meniscus lesion and were also immortalised by a pGEM7/SV40 TAg vector construct. Several studies confirmed that both immortalised cell lines represent a valuable tool to study mechanisms that induce synoviocyte activation [11-13]. Both cell lines were cultured as monolayers in RPMI 1640 medium supplemented with 10% (v/v) foetal calf serum (FCS), 2 mM glutamine and 50 μg/mL penicillin-streptomycin.

### Stimulation protocols

**Figure 1:**
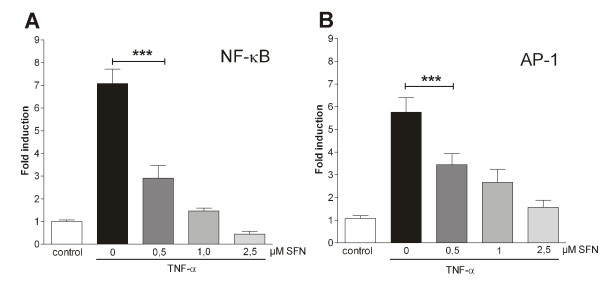
**Sulforaphane (SFN) inhibits TNF-α-induced activation of NF-κB and AP-1**. Synoviocytes (K4IM) were pre-treated (30 min) with increasing concentrations of SFN or solvent and stimulated with 10 ng/mL TNF-α for 6 h. Cells were transfected with p(NF-κB)_3 _**(A) **or p(AP-1)_5 _**(B) **and dual luciferase assay was measured as described in Wruck *et al*. 2007. Treatment groups were normalised to the control group (= 1). All experiments were performed with *n *= 8. Data represent mean + SEM. Statistical significance is marked as ***P < 0.001. (One-way ANOVA with Bonferroni multiple comparison post hoc test, GraphPad Prism 5 software; GraphPad Software, La Jolla, CA, USA.).

**Figure 2:**
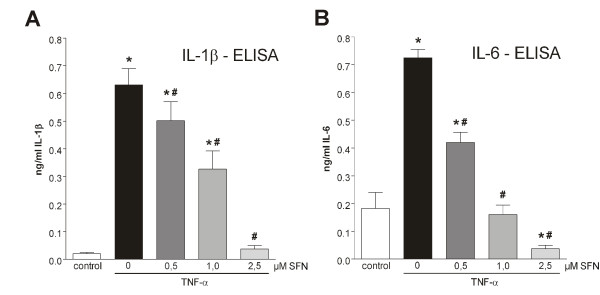
**Sulforaphane (SFN) inhibits TNF-α-induced production of IL-1β and IL-6**. Synoviocytes (K4IM) were pre-treated (30 min) with increasing concentrations of SFN and stimulated with 10 ng/mL TNF-α for 6 h. ELISA for IL-1β **(A) **and IL-6 **(B) **was measured as described in methods. Experiments were performed with *n *= 8. Graphs represent mean + SEM. Statistical significances are marked as *P < 0.05 vs. control, #*P *< 0.05 vs. TNF-α treated only. (One-way ANOVA with Bonferroni multiple comparison post hoc test, GraphPad Prism 5 software; GraphPad Software, La Jolla, CA, USA.).

**Figure 3:**
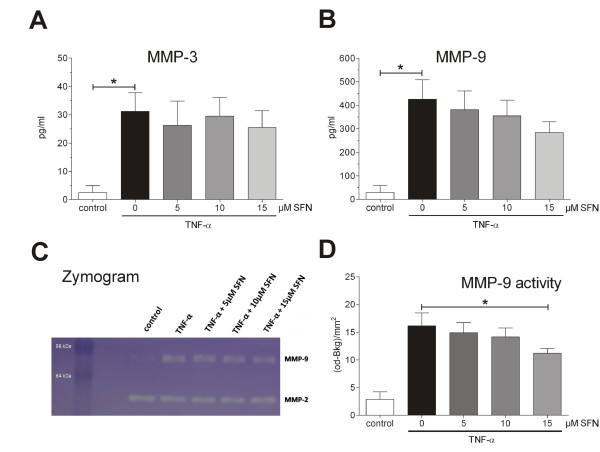
**Sulforaphane (SFN) downregulates neither matrix metalloproteinase (MMP) secretion nor catalytic activity**. Synoviocytes (HSE) were pre-treated 30 min with various concentrations of SFN and then stimulated with 10 ng/mL TNF-α for 6 h. Luminex multiplex assay for MMP-3 **(A) **and MMP-9 secretion level **(B) **was carried out as described above. The catalytic activity of MMP-9 was visualised by zymography **(C) **and quantified by densitometric analysis with ImageJ **(D)**. All experiments were performed with *n *= 3. Graphs represent mean + SEM. Statistical significance is marked as *P < 0.05. (One-way ANOVA with Bonferroni multiple comparison post hoc test, GraphPad Prism 5 software; GraphPad Software. La Jolla, CA, USA.).

We used K4IM cells for the analysis of the inflammatory response of synoviocytes (Figures 1 and 2). The examination of matrix metalloproteinase (MMP) expression and activity was conducted with HSE cells (Figure 3), because it is known that these cells express MMP-3 and -9. For these experiments, cells were pre-treated for 30 min with solvent or increasing concentrations of SFN, before they were stimulated with 10 ng/mL TNF-α to examine the inhibitory effect of SFN on NF-κB, AP-1 and the downstream cytokine and MMP expression.

**Figure 4:**
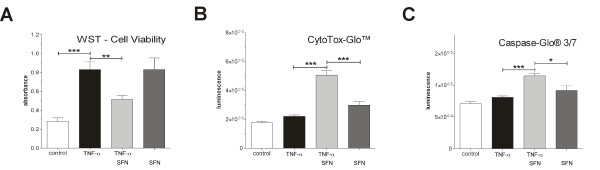
**Sulforaphane (SFN) induces apoptosis in TNF-α pre-treated 'transformed' synoviocytes**. HSE wild-type cells were either pre-treated (3 h) with 10 ng/mL TNF-α and then stimulated for a further 6 h with 6.25 μM SFN or only stimulated for 6 h with 6.25 μM SFN without TNF-α pre-treatment and WST **(A)**, CytoTox-Glo™ **(B) **and Caspase-Glo™ 3/7 **(C) **assays were performed as recommended by the manufacturers. All experiments were performed with *n *= 8. Graphs represent mean + SEM. Statistical significances are indicated as *P < 0.05, **P < 0.01, ***P < 0.001. (One-way ANOVA with Bonferroni multiple comparison post hoc test, GraphPad Prism 5 software; GraphPad Software, La Jolla, CA, USA.).

**Figure 5:**
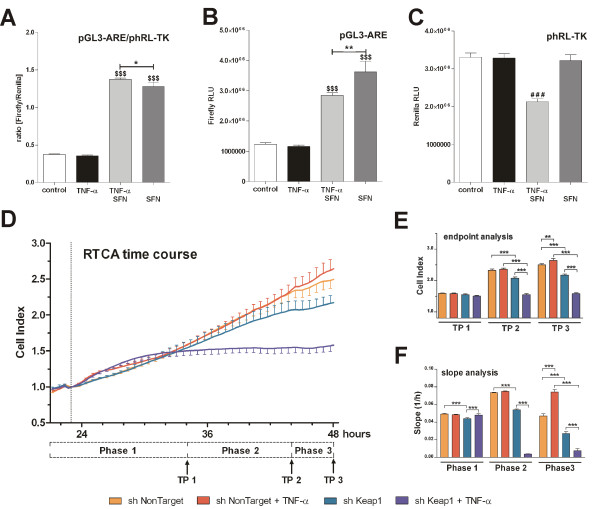
**Nrf2 activation leads to decreased proliferation of TNF-α pre-treated synoviocytes**. HSE cells were co-transfected with pGL3-ARE/phRL-TK and either pre-treated (3 h) with 10 ng/mL TNF-α and then stimulated for a further 6 h with 6.25 μM sulforaphane (SFN) or only stimulated for 6 h with 6.25 μM SFN without TNF-α pre-treatment. Afterwards, dual luciferase reporter gene (DLR) assay was carried out **(A-C)**. A real-time cell analysis (RTCA) experiment was performed with HSE cells stably transduced with either a shRNA targeting Keap1 mRNA or a shNonTarget control construct. Twenty-four h after seeding, the cells were treated with 10 ng/mL TNF-α and were observed for further 24 h by the RTCA device **(D)**. The Cell Index was determined at three time-points (TP) **(E) **and the slope of the curve was calculated for three characteristic phases of proliferation **(F)**. All experiments were performed with *n *= 8. Graphs represent mean + SD. Statistical significances are indicated as $*P *< 0.05, $$*P *< 0.01, $$$*P *< 0.001 vs. control; ###*P *< 0.001 vs. all other groups; *P < 0.05, **P < 0.01, ***P < 0.001 as indicated. (DLR: one-way ANOVA with Bonferroni multiple comparison post hoc test; RTCA analysis: two-way ANOVA with Bonferroni post hoc tests, GraphPad Prism 5 software; GraphPad Software, La Jolla, CA, USA.).

For experiments, which were performed to gain insights on the effects of SFN on already inflamed cells, the HSE cell line was used (Figures 4 and 5). These cells were pre-treated with 10 ng/mL TNF-α for 3 h to induce a proinflammatory state, before they were stimulated with 6.25 μM SFN for a further 6 h.

### Transient transfection of cell lines

For transient co-transfection with reporter gene plasmids together with the Renilla luciferase coding phRL-TK internal control plasmid, 4.0 × 10^6 ^cells were seeded in a 100 mm dish one day before transfection. Transfection was carried out with Lipofectamine^™ ^2000 reagent (Invitrogen, Darmstadt, Germany) according to the manufacturer's recommendations. After removal of the transfection mixture, cells were trypsinised and 2.5 × 10^4 ^cells per well were seeded in a 96-well microtitre plate for stimulation experiments.

### Stable Keap1 knockdown via lentiviral short hairpin RNA (shRNA) transduction

The real-time cell proliferation analysis (RTCA) experiment was performed with HSE cells, which were transduced with a pLKO.1 shRNA construct directed against Keap1 (MISSION^™ ^shRNA assortment, Sigma-Aldrich, Germany) by lentiviral gene delivery. In order to exclude variations due to the vector backbone and to prove that observed effects are exclusively dependent on the shRNA, we also transduced HSE cells with a shNonTarget control construct.

### Luciferase assays

After stimulation, cells were lysed in passive lysis buffer (Promega, Madison, WI, USA) and lysates were transferred to a white 96-well microtitre plate for luminescence measurement in a GloMax^™ ^luminometer (Promega, Madison, WI, USA). Dual-luciferase reporter gene assay (Promega, Madison, WI, USA) was performed as described in Wruck *et al*. 2007. The following constructs were used:

**p(NF-*κ*B)_3_-Luc **refers to a concatenated trimer of the wild-type sequence ATGT**GGGATTTTCC**CATG (the core sequence is bold). Both strands:

5'-CATGTGGGATTTTCCCATGAGTGAGGGGACTTTCCCAGGCATGTGGGATTTTCCCATGG-3' and

5'-CTAGCCATGGGAAAATCCCACATGCCTGGGAAAGTCCCCTCACTCATGGGAAAATCCCACATGGTAC-3'.

**pAP-1_5_-Luc **refers to a concatenated pentamer of the AP-1 consensus sequence TGAGTCA.

**pGL3-ARE **refers to the antioxidant response element (ARE) consensus sequence of the rat NAD(P)H dehydrogenase, quinone 1 (NQO1) promoter region, as described in [14].

All strands were cloned in the pGL3-promoter vector (Promega, Madison, WI, USA). The luciferase assay was carried out as described in [15].

### Enzyme-linked immunosorbent assay (ELISA)

The concentrations of IL-1β and IL-6 in the synoviocyte culture supernatants were determined by ELISA. The assay was performed according to the manufacturer's instructions.

### Luminex xMAP^™ ^multiplex assay

The total levels of secreted MMP-3 and MMP-9 were quantified by Luminex xMAP^™ ^technology in duplicates. For that purpose, HSE culture supernatants were used after experimental treatment. The Fluorokine^™ ^MAP human MMP base kit (Catalogue No. LMP000) was purchased from R&D Systems (Minneapolis, MN, USA) and utilised as recommended by the manufacturer.

### Zymographic analysis

Zymographic analysis was used to determine the activity of MMP-9 after secretion into the cell culture medium over a fixed period of time. The method was performed according to a standard procedure described previously [16]. Briefly, following 24 h of experimental treatment, cell-conditioned media were harvested and concentrated for zymography. Total protein concentrations were determined by BCA (bicinchoninic acid) protein assay. Equal concentrations of total protein were used for analysis. Therefore, a total sample volume of 15 μL was loaded on a 12.5% PAGE gel containing 0.1% gelatin (BioRad, Munich, Germany). Gels were run for 90 min at 150 volts and were then incubated in 2.5% Triton-X (Merck, Darmstadt, Germany) for 1 h at room temperature (RT) on a rocker. In the next step, they were washed with distilled water and incubated overnight in renaturation buffer (10 mM Tris HCl, 1 mM CaCl_2 _× 2 H_2_O, 40 mM NaCl, 0.2 μM ZnCl_2_, pH 7.5) at 37°C. Gels were briefly washed and stained (1 g Coomassie blue, 200 mL methanol, 50 mL acetic acid, 250 mL H_2_O) for 1 h at RT on a rocker, followed by destaining in destaining buffer (200 mL methanol, 50 mL acetic acid, 250 mL H_2_O) until bands were clearly visible. Gels were scanned for densitometry analysis by ImageJ software (Wayne Rasband, National Institutes of Health, Bethesda, MD, USA). As MMP-2 is constitutively expressed and thus not regulated, its band served as an internal loading control for normalisation of densitometric data on MMP-9.

### Cell viability assay

A 4-[3-(4-iodophenyl)-2-(4-nitrophenyl)-2H-5-tetrazolio]-1,3-benzene disulfonate (WST) assay was used to measure cell viability. Therefore the cells were seeded in 96-well plates. After treatment with or without TNF-α [10 ng/mL] for 3 h, cells were treated with 6.25 μM SFN for a further 6 h. After the stimulation, the media were supplemented with 10 μL/well (96-well plate) WST 2 h before spectrophotometric evaluation. Conversion of WST to formazan was measured at 450 nm by microplate spectrophotometry (Tecan Sunrise™, Männedorf, Switzerland). This reaction reflects the reductive capacity of the cells, thereby representing the viability.

### Cytotoxicity assay

CytoTox-Glo™ cytotoxicity assay (Promega, Madison, WI, USA) was used to measure cytotoxicity. Cells were seeded in 96-well plates and after treatment with or without TNF-α [10 ng/mL] for 3 h, cells were treated with 6.25 μM SFN for a further 6 h. A CytoTox-Glo™ cytotoxicity kit was used according to the manufacturer's recommendation. Luminescence was measured in a 96-well plate reader (GloMax™ 96 microplate luminometer; Promega, Madison, WI, USA).

### Quantification of caspase activities

For measurements of caspase 3 and 7 activity, the Caspase-Glo^™ ^3/7 assay (Promega, Madison, WI, USA) was carried out according to the manufacturer's instructions. Cells were treated as described for the WST and CytoTox-Glo™ assay. This assay is based on the cleavage of the DEVD sequence of a luminogenic substrate by caspases 3 and 7, which results in a luminescent signal.

### Real-time cell analysis (RTCA)

We analysed the effect of Nrf2 and TNF-α on proliferation of HSE cells by continuous real-time impedance measurements using the xCELLigence system (Roche Diagnostics, Mannheim, Germany). The xCELLigence system contains a 96-well cell culture plate with a microelectrode array in the bottom of each well (E-Plate). The electrode impedance of the separate wells can be measured in defined intervals and is finally converted to a dimensionless parameter named Cell Index (CI). The CI is zero without cell adherence and increases when cells attach or proliferate on the microelectrode surface. To measure the background impedance all wells of the E-Plate were filled with 200 μL of growth medium. Afterwards 5.0 × 10^3 ^HSE cells stably expressing a shRNA targeting Keap1 or a non-targeting control shRNA were seeded by replacing 100 μL of the total medium per well. Subsequently, impedance measurements were conducted at intervals of 20 min under normal cell culture conditions (37°C in a humidified 5% CO_2 _atmosphere). After an overnight adherence phase, TNF-α was added to the respective wells in a final concentration of 10 ng/mL by replacing 100 μL medium per well, whereas control cells received equal amounts of fresh medium. Impedance measurements were continued then for additional 24 h. Afterwards the CI was normalised to the time-point of TNF-α addition and analysed using the RTCA software version 1.2.1 (Roche Diagnostics). All measurements were conducted with eight replicates.

## Results

### SFN blocked TNF-α-induced NF-κB and AP-1 activation in synoviocytes

To analyse the NF-κB and AP-1 activation *in vitro*, we established a dual luciferase reporter gene assay utilising the *cis*-acting elements of NF-κB and AP-1. Synoviocytes (K4IM) were pre-treated for 30 min with increasing concentrations of SFN or solvent (0.25% DMSO) as control and stimulated with 10 ng/mL TNF-α for 6 h.

Stimulation with TNF-α activated the NF-κB-dependent luciferase gene expression 7-fold compared to the control. Pre-incubation with 0.5 μM SFN nearly halved the TNF-α-induced activation to 3-fold over control. Doubling the SFN concentration further halved the TNF-α-induced activation to 1.5-fold and pre-incubation with 2.5 μM SFN completely restrained the TNF-α-induced activation (Figure 1A).

Nearly the same effect of SFN could be seen with the *cis*-acting element of AP-1. The TNF-α-induced 6-fold induction of AP-1-dependent luciferase gene expression was reduced to 3.5-fold by pre-incubation with 0.5 μM SFN. Incubation with 1 μM SFN further reduced the activation to 2.5-fold. TNF-α-induced AP-1 activation was also completely blocked by 2.5 μM SFN (Figure 1B).

### SFN inhibited the production of proinflammatory cytokines in synoviocytes

We examined the effect of SFN on TNF-α-induced IL-1β and IL-6 secretion. Synoviocytes (K4IM) were stimulated with 10 ng/mL TNF-α for 24 h in the presence of 0, 0.5, 1.0, and 2.5 μM SFN and the concentration of IL-1β and IL-6 in the supernatant was measured via ELISA. SFN inhibited the TNF-α-induced release of IL-1β and IL-6 in a concentration-dependent manner and this effect was already significant at the lowest tested concentration of 0.5 μM (Figure 2A and 2B).

### SFN altered neither MMP secretion nor activity

One feature of RA is an overexpression and persistent activity of MMPs whereby the extracellular matrix (ECM) is destructed. We therefore studied the effect of SFN on MMP-3/9 in our model of pannus formation. TNF-α treatment significantly upregulated MMP-3/9 secretion and MMP-9 activity in cultured synoviocytes. MMP-3/9 secretion at low SFN concentration showed a trend of downregulation, which did not reach significance in our model (Figure 3A and 3B). However, a high concentration of 15 μM SFN significantly reduced the MMP-9 activity in this experimental design, probably due to toxicity (Figure 3C and 3D).

### SFN induced apoptosis in TNF-α-stimulated synoviocytes

We used WST to study the molecular mechanism by which SFN inhibits the proliferation of synoviocytes. CytoTox-Glo and Caspase-Glo assays (Promega) were used to analyse the effects of SFN on cell toxicity and apoptosis. The results demonstrate that neither TNF-α nor SFN treatment alone had any cytotoxic effects compared to untreated control cells (Figure 4B, second and fourth column). In contrast, the treatment with TNF-α or SFN alone led to an increased cell viability (Figure 4A, second and fourth column). Indeed, 6.25 μM SFN had a cytotoxic effect on TNF-α pre-treated but not on untreated cells (Figure 4B, third column). The caspase enzyme activity assay confirmed these findings. These results demonstrate that there were no effects due to single administration of TNF-α or SFN. However, a significant increase of caspase-3/7 activity was found after 6.25 μM SFN stimulation of TNF-α pre-treated cells (Figure 4C, third column).

### Nrf2/ARE-activity is crucial for the differential effects of the treatments

In order to investigate the effects of these treatments on Nrf2/ARE activity, we carried out a dual luciferase reporter gene assay with the ARE sequence of the rat NQO1 gene. We were able to show that SFN induces Nrf2/ARE activity (Figure 5A, fourth column), in contrast to TNF-α, which caused no difference in ARE-driven luminescence compared to untreated cells (Figure 5A, second column). Co-stimulation of TNF-α pre-treated synoviocytes seemed to further increase the SFN-mediated Nrf2/ARE activation (Figure 5A, third column), but analysis of the raw luminescence signals of the ARE-regulated firefly luciferase and the internal control Renilla luciferase revealed a TNF-α-dependent decrease in luminescence intensities in co-stimulated cells (Figure 5B and 5C, third column). There is evidence that the supposed increased ratio of co-stimulated cells is based on a decreased Renilla-mediated luminescence signal, resulting from a decreased amount of vital cells. DMSO, used as vehicle in a concentration of 0.25%, showed no significant effect on ARE activation (data not shown). We further aimed to examine whether Nrf2/ARE activation has an effect on growth and proliferation processes independent of the effects of SFN application. We also intended to investigate to which extent the Nrf2/ARE activity might be responsible for the observed effects on co-treated cells. Therefore, we stably transduced HSE cells with the pLKO.1 vector containing a shRNA targeting Keap1 (the inhibitor of Nrf2) to achieve an increased Nrf2 expression and activation, and performed a real-time analysis of cell proliferation. TNF-α stimulation initially showed no difference in proliferation between the experimental groups (Figure 5D and 5F, Phase 1). Comparing the untreated shKeap1 cell line with the untreated shNonTarget cells over the whole time, we only found a minor difference in terms of cell growth and proliferation. TNF-α treatment of shNonTarget cells resulted in increased proliferation compared to the untreated shNonTarget cells (Figure 5D, Phase 3), as indicated by a higher cell index (Figure 5E, TP 3 first and second column) and an increased proliferation rate (Figure 5F, TP 3 first and second column) after 24 h of stimulation time. The same TNF-α treatment showed the opposite effect in shKeap1 cells (Figure 5D und 5F, Phase 2). These cells exhibited a clearly decreased cell index (Figure 5E, TP 3 first column) and showed a lower proliferation rate already after 10 h of stimulation with no recovery from this state during the entire experiment.

## Discussion

Recently, we showed that oxidative stress is significantly involved in cartilage degradation in experimental arthritis and that Nrf2 activity is a major requirement for limiting cartilage destruction [8]. Other detrimental factors are unbounded inflammation and rampantly growing pannus, invading cartilage and bone. The data suggest that the proinflammatory transcription factors NF-κB and AP-1 mediate pannus formation via induction of synoviocyte proliferation and resistance against apoptosis [17].

The objective of this study was to find substances that activate Nrf2 to protect cartilage tissue from oxidative stress on the one hand and to inhibit the proinflammatory transcription factors NF-κB and AP-1 in order to attenuate inflammation and pannus progression on the other hand. Beside inflammatory processes, pannus formation is a main feature of RA [18]. Therefore, we used TNF-α-stimulated synoviocytes to model synovial hyperplasia as part of pannus formation with rampantly growing and cytokine producing cells.

Here we tested the anti-rheumatic potential of SFN *in vitro*. First data showed that SFN has anti-arthritic and immune regulatory effects in an *in vitro *and *in vivo *model of RA [19]. In addition, it has been shown that SFN induces apoptosis in cancer cells, and that it inhibits the cell cycle, angiogenesis and inflammation [6,20]. Contrary to that, SFN is also known to efficiently activate Nrf2 and thereby protect cells against oxidative damage [21]. Interestingly, in this context, loss of Nrf2 lead to significantly more cartilage damage in an *in vivo *model of RA [8]. Until now, no explanation for these differential effects of SFN on cells is described.

First, we investigated the expression level of the proinflammatory cytokine IL-1β in our model. TNF-α stimulation upregulated the secretion of IL-1β in the cell culture supernatant. Incubation with SFN markedly reduced this cytokine upregulation in a dose-dependent manner (Figure 2A). This may be a consequence of the reduced activation of the proinflammatory transcription factors NF-κB and AP-1 due to SFN treatment (Figure 1A and 1B).

Recently, we described IL-6 as a target gene of Nrf2 and SFN stimulation as an inductor of IL-6 expression in hepatocytes [22]. In synoviocytes, SFN failed to upregulate IL-6 (data not shown) and, in contrast, it significantly inhibited TNF-α-induced IL-6 upregulation (Figure 2B). However, the molecular mechanisms of these opposing effects of Nrf2 regarding IL-6 expression remain to be elucidated. Taken together, these data indicate that SFN acts anti-inflammatory in our *in vitro *RA model.

Besides cytokines, ECM-degrading matrix metalloproteinases (MMPs) are important in arthritis. MMPs are mainly regulated by an AP-1 and NF-κB promoter binding motif [23]. We therefore investigated the secretion levels of MMP-3 and -9 and the activity state of MMP-9 in our *in vitro *model of RA. We showed that TNF-α induces the secretion of MMP-3/9 as well as the activity of MMP-9. Although, co-treatment with SFN could neither downregulate the expression level of MMP-3 nor MMP-9 (Figure 3A-D).

There are several lines of evidence suggesting that cell cycle progression and transcription factor activation of synovial cells is altered towards a transformed 'tumour-like' manner in RA [17]. It has been demonstrated that activation of NF-κB and AP-1 protects cells in the synovium against apoptosis and provides a link between inflammation and hyperplasia. This gave first evidence that inhibition of NF-κB and AP-1 reduces viability and proliferation of inflamed synovial fibroblasts [17,24-27]. Based on this, we hypothesised that NF-κB and AP-1 activation is vital for cytokine-activated synoviocytes and, as a consequence, deactivation or inhibition of these factors via SFN results in cell death. First, we studied the effect of TNF-α stimulation on cultured synoviocytes. As described, TNF-α stimulation led to NF-κB and AP-1 activation (Figure 1) and cells reacted with enhanced cytokine production (Figure 2). SFN totally blocked the TNF-α-induced transformation of synoviocytes and, moreover, induced caspase activation (Figure 4C) and cell death (Figure 4A and 4B). In contrast, SFN treatment of naïve - not TNF-α-stimulated - synoviocytes resulted in Nrf2 activation (Figure 5A-C) and upregulation of cytoprotective enzymes such as HO-1, NQO1, thioredoxins and thioredoxin reductases. Furthermore, we were able to show that not only the SFN-mediated NF-κB inhibition but also a SFN-independent Nrf2 activation led to the same effect of decreasing cell viability, as in co-stimulated wild-type cells in the previous experiments. The RTCA experiments clearly revealed a Nrf2-driven decrease of proliferation in response to TNF-α in HSE cells stably transduced with shRNA directed against Keap1 (the inhibitor of Nrf2). It was already shown that Nrf2 activation leads to an upregulation of heme oxygenase-1 (HO-1) [28]. Heme oxygenase-1 produces carbon monoxide, which is described to efficiently inhibit NF-κB activity [29,30]. Hence, we suggest that the genetically increased Nrf2 activity via Keap1 silencing and the resultant HO-1-mediated CO production cause a similar inhibition of NF-κB as SFN does, leading to the same impaired outcome in cell viability and proliferation. The RTCA experiment emphasises that the observed effects of SFN on TNF-α pre-treated cells may not only be explained by direct inhibition of NF-κB but also by SFN-driven induction of Nrf2 activity, which indirectly inhibits NF-κB via HO-1-mediated CO production.

## Conclusions

These data give first evidence that SFN selectively harms 'transformed' synoviocytes but vitalises naïve 'untransformed' synoviocytes. Thus, since we have shown that Nrf2 deficiency results in exacerbated cartilage destruction, Nrf2 activation via SFN may protect healthy synoviocytes and may lead to improved cartilage regeneration. Due to the fact that the data is based on experiments done with immortalised cell lines, there is importance to validate these results with primary fibroblast-like synoviocytes (FLS) cultures.

Our results demonstrate that SFN has anti-phlogistic properties and may retard pannus formation. Moreover, SFN activates the cell protective transcription factor Nrf2 and may prevent cartilage destruction in RA. These findings indicate that treatment of RA patients with SFN might be considered as a novel therapeutic strategy to combat joint destruction and inflammation in RA.

## Abbreviations

AP-1: activator protein-1; ARE: antioxidant response element; ECM: extracellular matrix; ELISA: enzyme-linked immunosorbent assay; FCS: foetal calf serum; FLS: fibroblast-like synoviocytes; IL-1β: interleukin-1beta; IL-6: interleukin-6; MMP: matrix metalloproteinase; NF-κB: nuclear factor kappa-light-chain-enhancer of activated B cells; NQO1: NAD(P)H dehydrogenase, quinone 1; Nrf2: nuclear factor erythroid 2-related factor 2; PAGE: polyacrylamide gel electrophoresis; RA: rheumatoid arthritis; RTCA: real-time cell proliferation analysis; SFN: sulforaphane; shRNA: short hairpin RNA; TNF-α: tumour necrosis factor-alpha.

## Competing interests

There are no financial or other relationships that might lead to a conflict of interest.

## Authors' contributions

CJW, AF and KT designed the study. AF, JL, SM, MT, CR and KT conducted the study. AF, JL, SM, US, SS, LKR, MT and CR performed the data collection. CJW, AF, TP, SL and DV conducted the data analysis and interpretation. CJW, AF, JL and TP drafted the manuscript. AF, JL, SM, US, SS, LKR, MT, CR, KT, DV, SL, TP and CJW revised the manuscript content. All authors read and approved the final manuscript.
